# Utilization of local pressure devices in pain management during injections: scoping review

**DOI:** 10.1590/0034-7167-2023-0399

**Published:** 2024-07-29

**Authors:** Jefferson Wildes da Silva Moura, Aline de Souza Bitencourt, Thiago Lopes Silva, Andréia Cristina Feitosa do Carmo, Luciano Marques dos Santos, Patrícia Kuerten Rocha

**Affiliations:** I Universidade Federal de Santa Catarina. Florianópolis, Santa Catarina, Brazil; II Universidade Federal de São Paulo. São Paulo, São Paulo, Brazil; III Universidade Estadual de Feira de Santana. Feira de Santana, Bahia, Brazil

**Keywords:** Equipment and Supplies, Injections, Pain, Nursing Care, Nursing, Tecnología de Bajo Costo, Inyecciones, Dolor, Atención de Enfermería, Enfermería

## Abstract

**Objective:**

to map scientific evidence regarding the use of local pressure devices in pain relief during injection procedures in patients.

**Methods:**

scoping review, following the recommendations of the JBI Manual for Evidence Synthesis and PRISMA-ScR, with searches conducted in the PubMed, EMBASE, CINAHL, LILACS, and PsycINFO databases, without temporal restrictions and with a cutoff date of March 2023.

**Results:**

a total of 1,514 studies were identified, with 20 articles included in the final sample. The ShotBlocker^®^ device was utilized during subcutaneous and intramuscular injections in children and adults, proving beneficial in reducing pain, anxiety, and fear associated with the procedure.

**Final considerations:**

the ShotBlocker^®^ is a low-cost, easy-to-use device that can enhance nursing clinical practice during painful procedures. However, studies involving the Brazilian Pikluc^®^ device are scarce. Further research involving both local pressure devices is recommended.

## RESUMEN

**Objetivo:**

mapear evidencia científica sobre el uso de dispositivos de presión local en el alivio del dolor durante la aplicación de inyecciones en pacientes.

**Métodos:**

revisión de alcance, siguiendo las recomendaciones del Manual JBI para Síntesis de Evidencia y PRISMA-ScR, con búsqueda en las bases de datos PubMed, EMBASE, CINAHL, LILACS y PsycINFO, sin restricciones temporales y con fecha límite hasta marzo de 2023.

**Resultados:**

se identificaron un total de 1,514 estudios, con 20 artículos incluidos en la muestra final. El dispositivo ShotBlocker^®^ se utilizó durante las inyecciones subcutáneas e intramusculares en niños y adultos, demostrando ser beneficioso en la reducción del dolor, la ansiedad y el miedo asociados con el procedimiento.

**Consideraciones finales:**

el ShotBlocker^®^ es un dispositivo de bajo costo y fácil manejo que puede mejorar la práctica clínica de enfermería durante procedimientos dolorosos. Sin embargo, los estudios sobre el dispositivo brasileño Pikluc^®^ son escasos. Se recomienda realizar estudios con ambos dispositivos de presión local.

## INTRODUCTION

During healthcare, patients are susceptible to different types of injections^(1)^, including subcutaneous, intradermal, and intramuscular. As these procedures are considered painful, they contribute to a stressful and unpleasant experience with psychological, physiological, and emotional repercussions^(2-3)^.

Therefore, it is essential to consider and apply interventions that reduce the pain associated with injections, aiming for better individual acceptance and a less traumatic experience^(1)^. The nurse, as the one responsible for medication administration, is the ideal professional to utilize available technologies for pain management^(4-5)^ during the procedure.

According to the International Association for the Study of Pain, pain is a sensory and/or emotional experience that causes discomfort to the individual, which can be associated with or similar to actual damage or potential tissue damage^(6)^. Indeed, it is recognized as the fifth vital sign and is directly related to the quality of healthcare provided by services, according to The Joint Commission^(7)^.

Despite the increase in studies aiming to understand the pathophysiology of pain, resulting in improvements in treatments and control, as well as in instruments and scales for its measurement, it is observed that pain management is still insufficient^(8-9)^, especially during specific procedures such as injections.

However, it is observed that healthcare technologies are being developed to make needle-related procedures less painful and traumatic^(10)^. Among the available technologies for managing pain during injections are vibration, application of cold or heat, muscle relaxation, virtual reality, audiovisual distraction, and local pressure devices, such as ShotBlocker^®^ and Pikluc^®(10-12)^.

Local pressure devices are innovations aimed at relieving pain and anxiety associated with injections, thus improving patients’ experience with the procedure. ShotBlocker^®^ and Pikluc^®^ are similar local pressure devices, developed to minimize pain during needle procedures^(11-12)^.

Both ShotBlocker^®^ and Pikluc^®^ are composed of small tips that, upon contact with the individual’s skin, sensitize the nerve endings at the injection site, distributing the impact of needle insertion and leading to pain reduction due to the mechanism described by the Gate Control Theory for pain control^(11-12)^.

Pikluc^®^ was developed in Brazil^(12)^ and has a butterfly shape, aiming to be more attractive to the pediatric population, while ShotBlocker^®^ was developed by American pediatrician James Huttner and was first used in 2002, in a pediatric clinic located in the United States of America (USA)^(13)^. It is worth noting that in a study conducted at the mentioned clinic, Maumee Pediatric Associates, 82 guardians of newborns, children, and adolescents who received injections were questioned about their child’s experience with the ShotBlocker^®^ device^(13)^. It was found that 61% of the guardians judged that their child was less worried during the procedure, 85% would like their child to use ShotBlocker^®^ in future interventions, and 87% would recommend the device^(13)^.

Regarding Pikluc^®^, a satisfaction survey was conducted with 143 individuals aged between five and 60 years or older who used the device during an influenza vaccination campaign in April 2019 in Paraná^(14)^. Of these, 90.2% of the participants reported a reduction in needle prick pain when using Pikluc^®^. Regarding pain, 48% of participants did not feel it, 43% reported mild pain, and 9% reported moderate pain. Regarding the effectiveness of Pikluc^®^, 20% of participants indicated that it reduced only pain, 22% that it reduced fear and anxiety, 48% that it reduced pain, fear, and anxiety, while 10% were unable to respond. It is worth noting that 95.1% of participants would use Pikluc^®^ again, and 95.8% would recommend it^(14)^.

ShotBlocker^®^ and Pikluc^®^ are immediate-effect devices, easy to use/handle, and cheaper than sprays, anesthetic creams, and vibratory distractions^(11-12)^. Although both were developed for the pediatric population, a quasi-experimental controlled study conducted with ShotBlocker^®^ in adults found that it was also effective in reducing pain associated with intramuscular injection in this population^(8)^. Regarding Pikluc^®^, no experimental studies were found using it, regardless of age group.

With the aim of providing scientific evidence on the use of local pressure devices during subcutaneous, intradermal, and intramuscular injections, the following question arose: What evidence exists regarding the use of local pressure devices in pain relief during injection procedures in patients?

## OBJECTIVE

To map scientific evidence regarding the use of local pressure devices in pain relief during injection procedures in patients.

## METHODS

### Ethical Considerations

This study is a scoping review, and evaluation by the Research Ethics Committee involving human subjects is waived.

### Study Type

This study is a scoping review aimed at exploring and mapping scientific evidence within a specific field, as well as identifying key knowledge gaps on the topic^(15)^, with the goal of enhancing practice. To conduct this review, the guidelines of the Joanna Briggs Institute (JBI) Manual for Evidence Synthesis were followed^(15)^.

### Methodological Procedure

The protocol for this research was registered on the Open Science Framework (OSF) platform, online, on July 13, 2023 (https://osf.io/weuf5/). It is worth noting that the parameters of the Preferred Reporting Items for Systematic Reviews and Meta-Analyses extension for Scoping Reviews (PRISMA-ScR) were adhered to^(16)^ to describe the scientific evidence regarding the use of local pressure devices ShotBlocker^®^ and Pikluc^®^ in reducing pain associated with subcutaneous, intradermal, and intramuscular injections.

To develop this review, the steps recommended by the JBI Manual for Evidence Synthesis were followed^(15)^, including: 1) defining the objective and research question; 2) establishing inclusion and exclusion criteria; 3) defining the strategy for selecting and extracting data; 4) conducting the search, selection, and analysis of publications in databases; and 5) presenting and synthesizing the results^(15)^.

The population, concept, and context (PCC) mnemonic were utilized^(15)^, with P representing patients regardless of age, C indicating pain relief associated with subcutaneous, intradermal, and intramuscular injections, and C signifying the use of local pressure devices ShotBlocker^®^ and Pikluc^®^. This was done to construct the guiding question: what evidence is available on the use of local pressure devices ShotBlocker^®^ and Pikluc^®^ in pain relief associated with subcutaneous, intradermal, and intramuscular injections, irrespective of the age of the patients?

Original articles of various designs, such as descriptive, exploratory, experimental studies, quasi-experimental studies, primary studies, systematic reviews, and meta-analyses, in Portuguese, English, and Spanish languages were included, without temporal restriction, with a cutoff date until March 2023. Editorials, letters to the editor, abstracts, expert opinions, conference proceedings, and publications not addressing the objective of this study were excluded.

### Data Collection and Organization

The literature search was conducted in April 2023, across the PubMed, EMBASE, Cumulative Index to Nursing and Allied Health Literature (CINAHL), Literatura Latino-Americana e do Caribe em Ciências da Saúde (LILACS), and PsycINFO databases. In order to encompass the widest range of studies, appropriate descriptors for each database were chosen, along with the use of keywords. Chart 1 presents the search strategies, noting that they were constructed by a librarian with expertise in the healthcare field. To retrieve studies, filters developed by experts from the BMJ Knowledge Center^(17)^ and the InterTASC Information Specialists’ Sub-Group (ISSG)^(18)^ were applied. Additionally, the recommendations for search strategy development advocated by the Peer Review of Electronic Search Strategies (PRESS) were followed^(19)^.

**Chart 1:** Search Strategies in Databases, 2023

Database	Search Strategy
PubMed	*((“analgesia”[MeSH Terms] OR “analgesia”[All Fields] OR “analgesias”[All Fields] OR “pain relief”[Text Word] OR (“pain”[MeSH Terms] OR “pain”[All Fields]) OR (“anxiety”[MeSH Terms] OR “anxiety”[All Fields] OR “anxieties”[All Fields] OR “anxiety s”[All Fields]) OR (“fear”[MeSH Terms] OR “fear”[All Fields])) AND (equipment and supplies [tw] OR “ShotBlocker”[All Fields] OR “equipment design”[Text Word] OR “device”[Title] OR “Design”[Title])) AND (“inject”[All Fields] OR “injectability”[All Fields] OR “injectant”[All Fields] OR “injectants”[All Fields] OR “injectate”[All Fields] OR “injectates”[All Fields] OR “injected”[All Fields] OR “injectible”[All Fields] OR “injectibles”[All Fields] OR “injecting”[All Fields] OR “injections”[MeSH Terms] OR “injections”[All Fields] OR “injectable”[All Fields] OR “injectables”[All Fields] OR “injection”[All Fields] OR “injects”[All Fields] OR (“needle s”[All Fields] OR “needled”[All Fields] OR “needles”[MeSH Terms] OR “needles”[All Fields] OR “needle”[All Fields] OR “needling”[All Fields] OR “needlings”[All Fields]))*
EMBASE	*(‘equipment design’/exp OR ‘equipment design’ OR ‘product design’/exp OR ‘product design’ OR ‘shotblocker’) AND (‘injection’/exp OR ‘blood vessel injection’ OR ‘gluteal injection’ OR ‘injection’ OR ‘injection solution’ OR ‘injections’ OR ‘percutaneous injection’ OR ‘needle’/exp) AND (‘fear’/exp OR ‘fear’ OR ‘anxiety’/exp OR ‘analgesia’/exp OR ‘analgesia’ OR ‘analgesia’ OR ‘pain management’ OR ‘pain relief’ OR ‘sequential analgetic analgesia’ OR ‘surgical analgesia’ OR ‘pain’/exp OR ‘acute pain’ OR ‘deep pain’ OR ‘lightning pain’ OR ‘nocturnal pain’ OR ‘pain’ OR ‘pain response’ OR ‘pain syndrome’ OR ‘treatment related pain’)*
CINAHL LILACS PSYCINFO	*(analgesia OR pain relief OR pain OR anxiety OR fear) AND (injection OR needle) AND (equipment design OR devices OR design OR equipment and supplies OR shotblocker)*

### Data Analysis

The study selection process occurred in two stages. In the first stage, two independent reviewers utilized the Rayyan QCRI Online Tool^(20)^ to read titles and abstracts. If there was disagreement between the reviewers, a third reviewer was consulted to evaluate and decide on the inclusion or exclusion of the study. In the second stage, one reviewer separated the pre-selected studies and sent them in full to the other reviewers. Each reviewer independently assessed and decided to include or exclude the study, based on pre-established criteria and the review’s objective. If consensus was not reached, the third reviewer was consulted again.

The final selection included studies that investigated the use of local pressure devices in alleviating pain associated with subcutaneous, intradermal, and intramuscular injections across all age groups. The studies underwent descriptive analysis, using a data collection instrument developed by the authors to extract and delimit evidence. This instrument encompassed publication details (authorship, year, country), objectives, methodological characteristics (study design, sample, setting), route of administration, main findings, and study evidence level.

To classify the evidence level of the studies, the categorization recommended by the Agency for Healthcare Research and Quality (AHRQ)^(21)^ was applied.

To summarize the results concerning the use of local pressure devices in pain relief associated with subcutaneous, intradermal, and intramuscular injections, charts were employed, as per JBI recommendations, to enhance visualization.

## RESULTS

**Figure 1:**
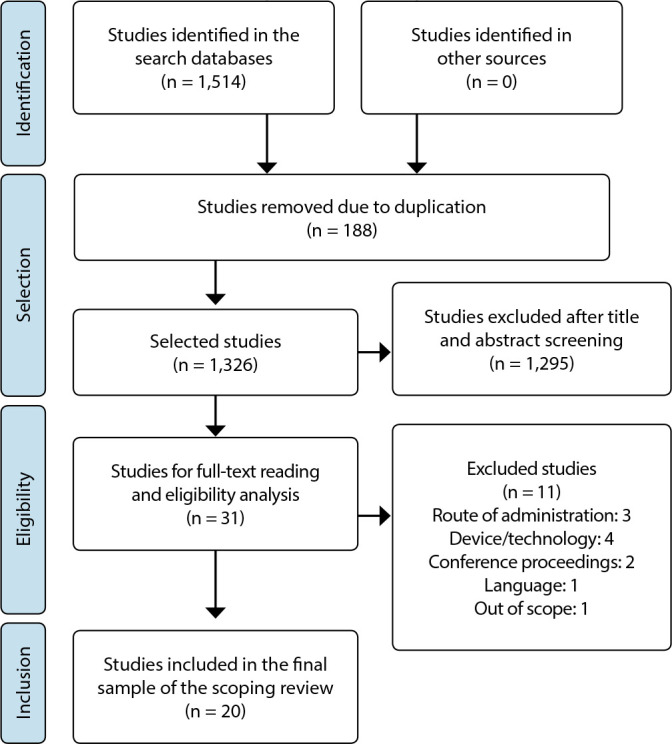
Flowchart of the article selection process for the review, 2023

*Source: Adapted from PRISMA^(22)^*

A total of 1,514 studies were found in the databases. During the initial assessment, 188 studies were excluded due to duplication, leaving 1,326 for title and abstract screening. At this stage, 1,295 studies were removed for not meeting the purpose of this review. Consequently, 31 studies were selected for full-text reading and eligibility analysis, with 11 studies being excluded. The final sample comprised 20 articles, and the process of identification, selection, eligibility, and inclusion is described in Figure 1, following the Preferred Reporting Items for Systematic Reviews and Meta-Analyses (PRISMA) flowchart^(22)^.

Among the 20 included articles, the oldest were published in 2009, and the most recent in 2023. Two (10%) were published in 2009, one (5%) in 2015, two (10%) in 2017, one (5%) in 2018, five (25%) in 2019, one (5%) in 2020, four (20%) in 2021, three (15%) in 2022, and one (5%) in 2023. Thus, 70% of the publications included in this review are from the last five years (2019 to March 2023).

Regarding the country of origin of the studies, Turkey led the research on injection pain relief with 16 (80%) studies, while the USA accounted for two (10%) studies, and the United Kingdom and India were responsible for one (5%) study each. Concerning the study design, eleven (55%) are clinical trials/experimental studies, four (20%) are prospective studies, two (10%) are quasi-experimental, two (10%) are systematic reviews, and one (5%) is a literature review.

Regarding the local pressure devices, 20 (100%) studies include the ShotBlocker^®^. It is worth noting that studies with Pikluc^®^ are scarce and were not retrieved from databases as they are not indexed materials. However, this device is part of the Brazilian reality and has been used in healthcare. Regarding the route of administration, 16 (80%) studies involve the intramuscular route, three (15%) involve the subcutaneous route, and one (5%) addresses both intramuscular and subcutaneous routes.

The studies included in this review are presented in Chart 2, containing information on authorship, year, country, objective, study design, sample, setting, route of administration, main results, and level of evidence.

**Chart 2:** Characteristics of the Studies Included in the Scoping Review, 2023

ID	Authorship Year Country	Objective	Study design Sample Setting Route of administration	Main results	Level of evidence
S1	Cobb *et al.* ^(23)^ 2009 United States of America	To examine the effectiveness of ShotBlocker^®^, a physical intervention designed to reduce injection pain in children.	Study type: Randomized Controlled Trial. Sample: 89 children, aged 4 to 12 years, and their respective parents. Setting: Pediatric clinic. Route of administration: Subcutaneous and intramuscular.	There was no evidence of ShotBlocker^®^ effectiveness in reducing pain associated with common childhood immunizations. Likewise, it was not effective in reducing childhood anxiety.	II
S2	Drago *et al.* ^(24)^ 2009 United States of America	To determine the efficacy of ShotBlocker^®^ in reducing pediatric pain during intramuscular injections.	Study type: Randomized Controlled Prospective Study. Sample: 165 children, aged 2 months to 17 years. Setting: Pediatric outpatient clinic of a University Hospital. Route of administration: Intramuscular.	Perceived pain scores by nurses and caregivers were lower in children who used ShotBlocker^®^ during immunization. However, this effectiveness was not as evident in self-reports from children aged 36 months or older regarding their pain. However, children aged 72 months or older reported pain relief when using ShotBlocker^®^.	II
S3	Çelik *et al.* ^(25)^ 2015 Turkey	To evaluate the effectiveness of ShotBlocker^®^ in reducing pain and anxiety associated with intramuscular injection in adults.	Study type: Randomized Placebo-Controlled Trial. Sample: 180 adults, aged 18 to 80 years. Setting: Outpatient clinic of a Public Hospital. Route of administration: Intramuscular.	Individuals who used ShotBlocker^®^ during intramuscular injection of sodium diclofenac (75 mg/3 mL) had significantly lower pain scores compared to individuals in the control and placebo groups. Therefore, ShotBlocker^®^ is recommended for relieving pain associated with intramuscular injection in adults. However, it did not reduce anxiety. Additionally, it may cause additional anxiety, so it is essential to introduce the device to individuals before use.	II
S4	Caglar *et al.* ^(26)^ 2017 Turkey	To examine the efficacy of ShotBlocker^®^ in controlling injection pain associated with the first intramuscular Hepatitis B vaccine administered to healthy full-term newborns.	Study type: Prospective Randomized Controlled Study Sample: 100 healthy full-term newborns. Setting: Ward of a private University Hospital. Route of administration: Intramuscular.	Pain scores from the Neonatal Infant Pain Scale (NIPS) at the time and post-injection were significantly lower in neonates who used ShotBlocker^®^ compared to the control group. Heart rate was lower in the intervention group three minutes after injection, but there was no significant difference in respiratory rate. ShotBlocker^®^ is recommended for relieving pain associated with intramuscular injection in healthy newborns.	II
S5	Emel *et al.* ^(27)^ 2017 Turkey	To examine the effects of ShotBlocker^®^ in relieving pain due to Hepatitis B vaccination in the deltoid muscle in adults.	Study type: Randomized Controlled Single-Blind Study. Sample: 242 individuals, aged 18 to 31 years. Setting: Nursing and Obstetrics Departments of a School of Health (University). Route of administration: Intramuscular.	There was no significant difference between the experimental and control groups regarding pain associated with Hepatitis B vaccine administered in the deltoid muscle. Therefore, ShotBlocker^®^ was not effective in pain reduction. However, it was observed that body mass index (BMI) interferes with pain perception, with pain decreasing as BMI increases.	II
S6	Canbulat Sahiner *et al.* ^(28)^ 2018 Turkey	To compare the effect of ShotBlocker^®^ and the combination of vibration and cold application (Buzzy^®^) in reducing pain during insulin administration in children.	Study type: Randomized Controlled Experimental Study. Sample: 60 children, aged 6 to 12 years. Setting: Department of Pediatric Endocrinology of a Medical School (University). Route of administration: Subcutaneous.	In perceived pain by caregivers and researcher/observer, as well as in children’s self-reports, children in the control group had higher pain scores compared to scores in the Buzzy^®^ and ShotBlocker^®^ groups. Anxiety scores of children in the Buzzy^®^ and ShotBlocker^®^ groups were lower compared to the control group. Children who used ShotBlocker^®^ had the lowest scores in both pain and anxiety.	II
S7	Aydin *et al.* ^(8)^ 2019 Turkey	To examine the effect of ShotBlocker^®^ in relieving pain associated with intramuscular injection.	Study type: Controlled Quasi-Experimental Study. Sample: 50 women, aged 18 to 45 years. Setting: Hospital inpatient unit. Route of administration: Intramuscular.	The experimental group had statistically significant lower pain scores compared to the control group. The use of ShotBlocker^®^ is recommended in intramuscular injection services and protocols and dissemination of its scientific evidence as a non-pharmacological method to promote its use in nursing clinical practice.	III
S8	Bilge *et al.* ^(29)^ 2019 Turkey	To compare the efficacy of ShotBlocker^®^ and cold spray in reducing pain related to intramuscular injection in adults.	Study type: Prospective Randomized Controlled Study. Sample: 120 adults, aged 18 years and older. Setting: Emergency Medicine Department of a Medical School (University). Route of administration: Intramuscular.	ShotBlocker^®^ was considered an effective non-pharmacological method in reducing pain related to intramuscular injection, with no difference in effectiveness compared to cold spray. However, administering the injection with ShotBlocker^®^ was more challenging compared to cold spray, according to participants. Further studies are recommended to facilitate the use of ShotBlocker^®^ in clinical practice.	II
S9	Sivri Bilgen *et al.* ^(30)^ 2019 Turkey	To investigate the effect of Buzzy^®^ and ShotBlocker^®^ in reducing pain induced by intramuscular penicillin injections in children.	Study type: Randomized Controlled Study. Sample: 150 children, aged 7 to 12 years. Setting: Pediatric Emergency Clinic. Route of administration: Intramuscular.	Children in the control group had significantly higher pain scores during penicillin injection than children in the ShotBlocker^®^ and Buzzy^®^ groups. The Buzzy^®^ group had the lowest pain scores compared to the ShotBlocker^®^ and control groups. However, ShotBlocker^®^ had lower scores compared to the control group, being a non-pharmacological option for relieving pain associated with intramuscular injection. Anxiety assessed before the procedure did not have a statistically significant difference between the groups.	II
S10	Yilmaz *et al.* ^(31)^ 2019 Turkey	To compare the effectiveness of Buzzy^®^, ShotBlocker^®^, and bubble-blowing in reducing pain and fear associated with intramuscular injection in children.	Study type: Prospective Randomized Clinical Trial. Sample: 160 children, aged 5 to 10 years. Setting: Hospital Pediatric Emergency Room. Route of administration: Intramuscular.	Pain and fear were significantly lower in children who used Buzzy^®^ compared to the control, ShotBlocker^®^, and bubble blowing groups. However, children in the ShotBlocker^®^ group also had lower pain and fear scores compared to the control and bubble blowing groups. Thus, ShotBlocker^®^ and Buzzy^®^ devices are recommended during intramuscular injection to reduce childhood pain and fear associated with the procedure.	II
S11	Şanlialp Zeyrek *et al.* ^(32)^ 2019 Turkey	Evaluate the effectiveness of physical-processual interventions in reducing pain during intramuscular injections.	Study type: Systematic Review and Meta-Analysis. Sample: 15 studies. Setting: Not applicable. Route of administration: Intramuscular.	The ShotBlocker^®^ is a tool to be considered for relieving pain associated with intramuscular injections.	I
S12	Inangil *et al.* ^(33)^ 2020 Turkey	Investigate the effect of mechanoneuroanalgesia and cold application on bruising, pain, and patient satisfaction for subcutaneous heparin injection.	Study type: Prospective Controlled Quasi-Experimental Clinical Research. Sample: 55 adults, aged 18 years and older. Setting: Orthopedics and Traumatology Wards of a University Hospital. Route of administration: Subcutaneous.	Both the use of mechanical analgesia through ShotBlocker^®^ and cold application reduced the pain associated with subcutaneous heparin injection. ShotBlocker^®^ was ineffective in reducing bruising but obtained a higher level of satisfaction with statistical significance.	III
S13	Ayinde *et al.* ^(34)^ 2021 United Kingdom	Review the effect of different intramuscular injection techniques on injection-associated pain in adults.	Study type: Systematic Review and Meta-Analysis. Sample: 29 studies. Setting: Not applicable. Route of administration: Intramuscular.	Contradictory evidence was observed regarding the effectiveness of ShotBlocker^®^, where one study shows a reduction in pain associated with intramuscular injection, while another study asserts that the pain was similar in the control and intervention groups. It is worth noting that the difference between the results may be related to a larger study population, smaller injection volume, and a healthier patient group.	I
S14	Kolcu *et al.* ^(35)^ 2021 Turkey	Investigate the effect of ShotBlocker^®^ on pain levels, anxiety, and satisfaction in subcutaneous injection of patients with chronic spontaneous urticaria.	Study type: Randomized Controlled Clinical Trial. Sample: 90 individuals, aged 18 years and older. Setting: Dermatology Clinic of a University Hospital. Route of administration: Subcutaneous.	Regarding pain and anxiety levels, there was no statistically significant difference between the ShotBlocker^®^, placebo, and control groups in the assessment after the injection. Regarding satisfaction levels after injection, the ShotBlocker^®^ group had a statistically high outcome compared to the placebo and control groups. However, it is believed that ShotBlocker^®^ could be presented to patients as an option due to its easy and cost-effective use.	II
S15	Şahan *et al.* ^(36)^ 2021 Turkey	Determine the effects of ShotBlocker^®^ application during intramuscular injection administration in adult patients to provide evidence-based practice.	Study type: Literature Review with Meta-Analysis. Sample: 5 studies. Setting: Not applicable. Route of administration: Intramuscular.	Pain levels in the experimental group where ShotBlocker^®^ was used during intramuscular injection in adult patients were significantly different compared to the control group. As a result of the meta-analysis, it was found that the application of ShotBlocker^®^ in intramuscular injection in adult patients reduced the intensity of patients’ pain.	I
S16	Yildirim *et al.* ^(37)^ 2021 Turkey	Evaluate the effect of ShotBlocker^®^ on pain and satisfaction of intramuscular injection in adult emergency patients.	Study type: Randomized Clinical Trial. Sample: 74 individuals, aged 18 years and older. Setting: Hospital Adult Emergency Department. Route of administration: Intramuscular.	The level of pain in the ShotBlocker^®^ group was lower compared to the control group. Meanwhile, the satisfaction level in the ShotBlocker^®^ group was higher compared to the control group. In both cases, the difference was statistically significant.	II
S17	Gürdap *et al.* ^(38)^ 2022 Turkey	Compare the effects of cold spray and ShotBlocker^®^ on pain reduction in adults caused by intramuscular injection in the adult emergency department.	Study type: Randomized Controlled Clinical Trial. Sample: 195 individuals, aged 18 years and older. Setting: University Hospital Adult Emergency Department. Route of administration: Intramuscular.	Cold spray proved to be the best resource for managing pain associated with intramuscular injection. However, ShotBlocker^®^ is also an option for pain reduction, as it was effective when compared to both the control group and placebo groups.	II
S18	Savcı *et al.* ^(39)^ 2022 Turkey	Examine the effectiveness of ShotBlocker^®^ and local vibration on pain perception and satisfaction during intramuscular antibiotic injection.	Study type: Randomized Controlled Experimental Study. Sample: 100 individuals, aged 18 years and older. Setting: Hospital Adult Emergency Department. Route of administration: Intramuscular.	Local vibration application was more effective in reducing pain and increasing satisfaction during intramuscular antibiotic injection compared to the ShotBlocker^®^ and control groups. It is noted that pain scores were lower and satisfaction scores were higher in the ShotBlocker^®^ group compared to the control group.	II
S19	Zengin *et al.* ^(40)^ 2022 Turkey	Test a new non-pharmacological intervention to reduce needle-related pain in the pediatric emergency department.	Study type: Randomized Controlled Clinical Trial. Sample: 159 children, aged 7 to 10 years. Setting: Pediatric Emergency Department. Route of administration: Intramuscular.	In the perceived pain by caregivers and researcher/observer, as well as in the children’s self-reports, pain scores in the Palm Stimulator group were statistically and significantly lower compared to the ShotBlocker^®^ and control groups. Although the perception of pain levels in the ShotBlocker^®^ group was lower than in the control group, this difference was not statistically significant. Regarding pre-procedural fear, there was no difference between the groups.	II
S20	Cmc *et al.* ^(41)^ 2023 India	To synthesize the best available research evidence regarding the effectiveness of physical stimulation for reducing injection pain in adults receiving intramuscular injections.	Study type: Systematic Review and Meta-analysis. Sample: 25 studies. Setting: Not applicable. Route of administration: Intramuscular.	ShotBlocker^®^ is effective in reducing pain associated with intramuscular injection. However, it is observed that the device may increase patients’ anxiety levels. It is reinforced that it is a promising technique in the management of pain associated with intramuscular injection, with low cost, and can be easily taught/learned.	I

*ID = Study Identification.*

## DISCUSSION

This study contributes to advancing research on the use of local pressure devices ShotBlocker^®^ and Pikluc^®^ during injections, aiming to reduce the pain, anxiety, and fear associated with these procedures. However, it is noted that upon searching the databases, the results found both nationally and internationally only include the ShotBlocker^®^ device, and the only study conducted with Pikluc^®^ so far was not retrieved from the databases. Nevertheless, the results evidenced in this review indicate converging and diverging points regarding the benefits of using the ShotBlocker^®^ device during the administration of intramuscular and subcutaneous injections in patients in the context of healthcare assistance.

During the neonatal period, the newborn undergoes different painful procedures, including the Hepatitis B vaccine administered intramuscularly (S4)^(26)^. The phenomenon of pain in Neonatology is subjective and complex, and since verbal reporting by the newborn is impossible, it is necessary to consider behavioral and physiological parameters to obtain a multidimensional assessment of pain^(42)^. When using the ShotBlocker^®^ device in healthy full-term newborns, a positive effect is observed in reducing vaccination-associated pain, as assessed by the Neonatal Infant Pain Scale (NIPS), as well as a systemic effect by reducing the heart rate of the neonate. However, the ShotBlocker^®^ did not interfere with the respiratory rate of the newborns^(26)^.

The ShotBlocker^®^ is a device available to Nursing, easily accessible, and with an affordable cost that has been shown to be effective in reducing pain in neonates. However, it is recognized that heart rate represents a physiological response, while the NIPS scale provides a behavioral response to pain and not its intensity. Additionally, this was the first needle procedure experienced by the participating newborns, and it is known that the response to pain is also related to past experiences (S4)^(26)^.

In Pediatrics, the ShotBlocker^®^ was used in different age groups, ranging from two months to 17 years old, and administration routes included intramuscular and/or subcutaneous (S1, S2, S6, S9, S10, S19)^(23-24,28,30-31,40)^. When evaluating children’s pain during routine immunization via intramuscular injection, using the Baker-Wong FACES Scale, it was found that the self-report of children between 36 and 71 months of age did not show a positive effect of the ShotBlocker^®^ device. However, when evaluating the self-report of children aged 72 months or older, it is noted that the device was useful in reducing pain associated with intramuscular injection, corroborating with the evaluation of caregivers and nurses who accompanied the procedure (S2)^(24)^. Although other parameters that permeate pain were not considered and evaluated.

During the administration of intramuscular penicillin in children between seven and 12 years old, the ShotBlocker^®^ and Buzzy^®^ devices were compared (S9)^(30)^. Buzzy^®^ is a device that combines vibration with the application of ice and is recommended during needle procedures^(43)^. To measure pain, the Visual Analog Scale (VAS) and Faces Pain Scale-Revised (FPS-R) were used, and before the procedure, children’s anxiety was measured using the State-Trait Anxiety Inventory for Children (STAIC)^(30)^. Regarding anxiety, there was no difference between the groups, but regarding pain, it was observed that Buzzy^®^ showed the best results, although ShotBlocker^®^ also proved to be effective when compared to the control group (S9)^(30)^.

When comparing the ShotBlocker^®^, Buzzy^®^, and bubble-blowing for relieving pain associated with intramuscular injection in children aged five to ten, as measured by Oucher pain scores^(31)^, it was observed that Buzzy^®^ yielded the best results, as evidenced in the previous study. However, the ShotBlocker^®^ showed lower pain scores compared to bubble-blowing and the control group. This result was also noted in the assessment of children’s fear using the Children’s Fear Scale (S10)^(31)^. In summary, although Buzzy^®^ is more effective in reducing pain and fear, the ShotBlocker^®^ remains an option for reducing pain and fear associated with intramuscular injections. It is emphasized that the mechanisms of action of the devices are also different.

Another device compared to the ShotBlocker^®^ in Pediatrics was the Palm Stimulator^(40)^. In this study, children aged seven to ten undergoing intramuscular injection were evaluated for pain using the VAS and FPS-R scales, and fear was assessed using the Children’s Fear Scale. Pain assessed by the child, caregiver, and researcher obtained lower scores in the Palm Stimulator group^(40)^. However, unlike other studies, the ShotBlocker^®^ did not show a statistically significant difference compared to the control group, despite having lower pain scores. Regarding fear, assessed before the procedure, there was no difference between the groups. It is noteworthy that this data is relevant for pain assessment, as fear can interfere with the child’s response (S19)^(40)^.

Regarding the subcutaneous route in children, the ShotBlocker^®^ was tested in two studies (S1, S6)^(23,28)^. In the study conducted during the administration of vaccines via subcutaneous and intramuscular routes in children aged four to twelve, it was observed that the ShotBlocker^®^ was not effective in reducing child pain and anxiety^(23)^. Child pain was assessed using self-report via the FPS-R scale, while caregivers and researchers used the VAS scale. Caregivers and researchers also observed the behavior/distress exhibited by the children before, during, and after the procedure, in addition to evaluating anxiety using the VAS scale (S1)^(23)^. It is emphasized that this scale was not developed for the purpose of assessing anxiety, but pain.

In contrast to previous studies, when comparing the ShotBlocker^®^ and Buzzy^®^ during insulin administration in children aged six to twelve diagnosed with Type 1 Diabetes Mellitus, the ShotBlocker^®^ obtained the lowest scores in both pain and anxiety assessment, based on self-report, caregiver, and researcher evaluations^(28)^. However, Buzzy^®^ was also effective when compared to the control group. In this study, an interview and observation form were used, in addition to the Children’s Anxiety and Pain Scale (CAPS) and FPS-R scales (S6)^(28)^. It is emphasized that this result may be related to the route of administration, as the subcutaneous route requires a simpler and less painful technique compared to intramuscular injection^(44)^.

In the context of adult care, the ShotBlocker^®^ was also evaluated during subcutaneous medication administration, involving medications such as heparin (S12)^(33)^ and omalizumab (S14)^(35)^. A study aimed to compare the effect of the ShotBlocker^®^ and cold application during heparin administration in the abdominal region in patients aged 18 and older^(33)^. The VAS scale was used to measure pain and patient satisfaction, along with a tool to assess bruising. It was observed that the ShotBlocker^®^ yielded the best results in terms of pain and satisfaction; however, it had no effect on bruising. It is emphasized that the lower the pain felt by the patient, the greater their satisfaction (S12)^(33)^.

The ShotBlocker^®^ was effective during subcutaneous injection administration, both in pediatric and adult populations. However, despite similar results, it is noteworthy that the study conducted with children utilized the Buzzy^®^ device, which combines cold with vibration (S6)^(28)^, while only cold application was used with adults (S12)^(33)^.

During subcutaneous omalizumab administration in individuals aged 18 and older, it was noted that the ShotBlocker^®^ did not show a statistically significant difference in reducing pain and anxiety compared to the control and placebo groups, with the latter using the smooth surface of the device^(35)^. However, participants were more satisfied with the device’s use. Similar to the previous study, the VAS scale was used to assess pain and satisfaction, along with the State-Trait Anxiety Inventory Form for anxiety measurement (S14)^(35)^. There is variation in the ShotBlocker^®^’s effect on reducing pain during subcutaneous injection, which may be related to different injection sites and substances administered.

Regarding the use of the ShotBlocker^®^ in adults during intramuscular injection administration, a greater number of studies are observed (S3, S5, S7, S8, S16, S17, S18)^(8,25,27,29,37-39)^. The ShotBlocker^®^ was effective in reducing pain during the administration of diclofenac 12 hours post-surgery (S7)^(8)^ and sodium diclofenac (S3, S16)^(25,37)^. However, a similar study found that cold spray was more effective in reducing pain during sodium diclofenac administration than the ShotBlocker^®^ (S17)^(38)^. Similarly, local vibration was more effective during antibiotic administration (S18)^(39)^. However, the ShotBlocker^®^ remains a viable option for pain management according to these same studies (S17, S18)^(38-39)^.

In contrast to findings in a previous study, there was no statistically significant difference in pain reduction when using the ShotBlocker^®^ or cold spray during sodium diclofenac administration^(29)^. Moreover, in this study, it was found that professionals experienced difficulty in administering intramuscular injections using the ShotBlocker^®^ (S8)^(29)^. Only one study in the adult population stated that the ShotBlocker^®^ is not effective in reducing pain, with the scenario being the administration of the Hepatitis B vaccine in individuals aged 18 to 31 years (S5)^(27)^, contradicting the results found in Neonatology (S4)^(26)^.

The VAS scale was used to measure pain (S3, S5, S7, S8, S16, S17, S18)^(8,25,27,29,37-39)^ and patient satisfaction (S16, S18)^(37,39)^, while the State-Trait Anxiety Inventory Form aimed to assess anxiety (S3)^(25)^. Regarding patient satisfaction with the use of the ShotBlocker^®^ during intramuscular injection, a positive response is observed (S16, S18)^(37,39)^. However, in one study, it was found that the device may increase the patient’s anxiety level, but it is still recommended as a resource to be implemented in clinical practice (S3)^(25)^.

### Study limitations

This research has significant limitations, including the absence of studies involving the Brazilian device Pikluc^®^, the lack of research on the devices’ use in intradermal injection, the absence of cross-references that could have been included in the sample, and the potential loss of studies due to databases not utilizing controlled descriptors.

### Contributions to Nursing

The findings of this review make important contributions by facilitating discussions about pain, anxiety, and fear management during subcutaneous and intramuscular injections in both pediatric and adult populations. Additionally, they contribute to disseminating knowledge about local pressure devices, their benefits and limitations, and support the enhancement of nursing care in clinical practice during the execution of painful and precise procedures.

## FINAL CONSIDERATIONS

The ShotBlocker^®^, a local pressure device, is accessible to nursing during painful procedures such as subcutaneous and intramuscular injections. It is observed that this resource, with its low cost and easy handling, can effectively manage pain, anxiety, and fear in pediatric and adult patients alike. Although there is considerable research demonstrating the efficacy of ShotBlocker^®^ in clinical settings, there are still discrepancies in the literature regarding its effectiveness. Therefore, it is crucial to conduct controlled clinical studies considering variables such as age, body mass index, medication, needle gauge, among others, and to compare them with other devices using the same mechanism of action. Additionally, it is advisable to conduct studies nationally and internationally with the Pikluc^®^ device, as well as research exploring the use of both devices in the intradermal route.
