# Supplementary material for: Utilization of local pressure devices in pain management during injections: scoping review
Source: Rev Bras Enferm. 2024 Jul 29;77(3):e20230399. doi: 10.1590/0034-7167-2023-0399 (PMC11290735; doi:10.1590/0034-7167-2023-0399)
Supplement: Translation

# Translation: Utilização de dispositivos de pressão local no manejo da dor durante injeções: revisão de escopo

**DOI:** 10.1590/0034-7167-2023-0399pt

**Authors:** Jefferson Wildes da Silva Moura, Aline de Souza Bitencourt, Thiago Lopes Silva, Andréia Cristina Feitosa do Carmo, Luciano Marques dos Santos, Patrícia Kuerten Rocha

**Affiliations:** I Universidade Federal de Santa Catarina. Florianópolis, Santa Catarina, Brasil; II Universidade Federal de São Paulo. São Paulo, São Paulo, Brasil; III Universidade Estadual de Feira de Santana. Feira de Santana, Bahia, Brasil

**Keywords:** Tecnologia de Baixo Custo, Injeções, Dor, Cuidados de Enfermagem, Enfermagem

## Abstract

**Objetivo:**

mapear evidências científicas acerca da utilização de dispositivos de pressão local no alívio da dor durante a aplicação de injeções em pacientes.

**Métodos:**

revisão de escopo, conforme recomendações do *JBI Manual for Evidence Synthesis* e PRISMA-ScR, com busca nas bases PubMed, EMBASE, CINAHL, LILACS e PsycINFO, sem recorte temporal e data limite até março de 2023.

**Resultados:**

foram encontrados 1.514 estudos, sendo incluídos 20 artigos na amostra final. O dispositivo ShotBlocker^®^ foi utilizado durante a aplicação de injeções subcutânea e intramuscular em crianças e adultos, sendo benéfico na redução da dor, da ansiedade e do medo associados ao procedimento.

**Considerações finais:**

o ShotBlocker^®^ é um dispositivo de baixo custo e fácil manuseio a ser utilizado para aprimorar a prática clínica da Enfermagem durante a realização de procedimentos dolorosos. Contudo, estudos com o dispositivo brasileiro Pikluc^®^ são escassos. Recomenda-se a realização de estudos com ambos os dispositivos de pressão local.

## INTRODUÇÃO

Durante o cuidado em saúde, os pacientes estão suscetíveis aos diferentes tipos de injeções^(1)^, incluindo a subcutânea, a intradérmica e a intramuscular. Por serem consideradas um procedimento doloroso, corroboram para que estejam associadas a uma experiência estressora e desagradável com repercussões psicológicas, fisiológicas e emocionais^(2-3)^.

Logo, é essencial que sejam pensadas e aplicadas intervenções que reduzam a dor associada às injeções, visando uma melhor aceitação do indivíduo, bem como uma vivência menos traumática^(1)^. O enfermeiro, por ser o responsável pela administração de medicamentos, é o profissional ideal para utilizar as tecnologias disponíveis para o manejo da dor^(4-5)^ durante a realização do procedimento.

Segundo a *International Association for the Study of Pain*, a dor é uma vivência sensorial e/ou emocional que causa desconforto no indivíduo, a qual pode ser associada ou similar ao dano real ou a um possível dano tecidual^(6)^. Inclusive, é reconhecida como o quinto sinal vital e está diretamente relacionada com a qualidade da assistência à saúde prestada pelos serviços, segundo a *The Joint Commission* ^(7)^.

Apesar do aumento de estudos que visam compreender a fisiopatologia da dor, que resultou na melhoria dos tratamentos e controle, assim como em instrumentos e escalas para a mensuração desta, observa-se que o seu manejo ainda é insuficiente^(8-9)^, especialmente durante a realização de procedimentos pontuais, como as injeções.

Contudo, observa-se que tecnologias em saúde estão sendo desenvolvidas para tornar os procedimentos que envolvem agulhas menos dolorosos e traumáticos^(10)^. Dentre as tecnologias disponíveis para manejar a dor durante a aplicação de injeções encontram-se a vibração, aplicação de frio ou calor, relaxamento muscular, realidade virtual, distração audiovisual e os dispositivos de pressão local, como, por exemplo, o ShotBlocker^®^ e o Pikluc^®(10-12)^.

Os dispositivos de pressão local são inovações que visam aliviar a dor e a ansiedade associadas às injeções e, consequentemente, melhorar a experiência dos pacientes com o procedimento. O ShotBlocker^®^ e o Pikluc^®^ são dispositivos de pressão local semelhantes, desenvolvidos para minimizar a dor durante a realização de procedimentos com agulhas^(11-12)^.

Tanto o ShotBlocker^®^ quanto o Pikluc^®^ são compostos por pequenas pontas que, ao entrarem em contato com a pele do indivíduo, sensibilizam as terminações nervosas do local da aplicação da injeção, além de distribuir o impacto da inserção da agulha, levando à redução da dor devido ao mecanismo descrito pela Teoria do Portão para controle da dor^(11-12)^.

O Pikluc^®^ foi desenvolvido no Brasil^(12)^ e possui formato de borboleta, visando ser mais atrativo para o público infantojuvenil, enquanto o ShotBlocker^®^ foi desenvolvido pelo médico pediatra norte-americano James Huttner, sendo utilizado pela primeira vez em 2002, numa clínica pediátrica localizada nos Estados Unidos da América (EUA)^(13)^. Cabe destacar que em estudo realizado na referida clínica, *Maumee Pediatric Associates*, 82 responsáveis por recém-nascidos, crianças e adolescentes que receberam injeções foram questionados acerca da experiência do seu filho com o dispositivo ShotBlocker^®(13)^. Constatou-se que 61% dos responsáveis julgaram que o filho ficou menos preocupado durante a realização do procedimento, 85% gostariam que seu filho utilizasse o ShotBlocker^®^ em intervenções futuras e 87% iriam recomendar o dispositivo^(13)^.

Acerca do Pikluc^®^, foi realizada uma pesquisa de satisfação com 143 pessoas com idade entre cinco e 60 anos ou mais que utilizaram o dispositivo durante uma campanha de vacinação contra a Gripe em abril de 2019 no Paraná^(14)^. Desses, 90,2% dos participantes perceberam redução na dor da picada da agulha ao utilizar o Pikluc^®^. Quanto à dor, 48% dos participantes não a sentiram, 43% indicaram dor leve e 9% indicaram dor moderada. Sobre a atuação do Pikluc^®^, 20% dos participantes indicaram que ele reduziu apenas a dor, 22% que reduziu o medo e a ansiedade, 48% que reduziu a dor, o medo e a ansiedade, enquanto 10% não souberam responder. Salienta-se que 95,1% dos participantes usariam o Pikluc^®^ novamente e 95,8% o recomendariam^(14)^.

O ShotBlocker^®^ e o Pikluc^®^ são dispositivos de efeito imediato, de fácil uso/manuseio, além de serem mais baratos que sprays, cremes anestésicos e distrações vibratórias^(11-12)^. Apesar de ambos terem sido desenvolvidos para o público infantil, estudo quase experimental controlado realizado com o ShotBlocker^®^ em adultos constatou que ele também foi eficaz na redução da dor associada à injeção intramuscular nesse público^(8)^. Quanto ao Pikluc^®^, não se encontrou estudos experimentais utilizando-o, independentemente da faixa etária.

Com a finalidade de fornecer evidências científicas sobre o uso dos dispositivos de pressão local durante a aplicação de injeções subcutânea, intradérmica e intramuscular, surgiu o seguinte questionamento: Quais evidências existem quanto à utilização de dispositivos de pressão local no alívio da dor durante a aplicação de injeções em pacientes?

## OBJETIVO

Mapear evidências científicas acerca da utilização de dispositivos de pressão local no alívio da dor durante a aplicação de injeções em pacientes.

## MÉTODOS

### Aspectos éticos

Trata-se de um estudo de revisão de escopo, sendo dispensada a apreciação pelo Comitê de Ética em Pesquisa envolvendo seres humanos.

### Tipo de estudo

Este estudo é uma revisão de escopo, que visa explorar e mapear as evidências científicas de determinado campo, assim como identificar as principais lacunas de conhecimento acerca da temática^(15)^, objetivando aprimorar uma prática. Para a realização desta revisão, foram seguidas as diretrizes do *Joanna Briggs Institute (JBI) Manual for Evidence Synthesis* ^(15)^.

### Procedimento metodológico

O protocolo desta pesquisa foi registrado na Plataforma *Open Science Framework* (OSF), online, em 13 de julho de 2023 (https://osf.io/weuf5/). Ressalta-se que foram seguidos os parâmetros do *Preferred Reporting Items for Systematic reviews and Meta-Analyses extension for Scoping Reviews* (PRISMA-ScR)^(16)^ visando descrever as evidências científicas acerca do uso dos dispositivos de pressão local ShotBlocker^®^ e Pikluc^®^ na redução da dor associada às injeções subcutânea, intradérmica e intramuscular.

Para o desenvolvimento desta revisão foram seguidas as etapas preconizadas pelo *JBI Manual for Evidence Synthesis* ^(15)^, a saber: 1) definição do objetivo e pergunta de pesquisa; 2) definição dos critérios de inclusão e exclusão; 3) definição da estratégia para seleção e extração dos dados; 4) realização da busca, seleção e análise das publicações nas bases de dados; e 5) apresentação e síntese dos resultados^(15)^.

Foi usada a mnemônica população, conceito e contexto (PCC)^(15)^, sendo: P: pacientes independente da faixa etária, C: alívio da dor associada a injeções subcutânea, intradérmica e intramuscular, C: uso dos dispositivos de pressão local ShotBlocker^®^ e Pikluc^®^; para a construção da pergunta norteadora: quais são as evidências disponíveis sobre a utilização dos dispositivos de pressão local ShotBlocker^®^ e Pikluc^®^ no alívio da dor associada a injeções subcutânea, intradérmica e intramuscular independentemente da idade dos pacientes?

Foram incluídos artigos originais de diferentes desenhos, como descritivo, exploratório, estudos experimentais, quase experimentais, estudos primários, revisões sistemáticas e meta-análises, nos idiomas português, inglês e espanhol, sem recorte temporal, com data limite até março de 2023. Foram excluídos os editoriais, cartas ao editor, resumos, opinião de especialistas, anais de congresso e as publicações que não respondessem ao objetivo desse estudo.

### Coleta e organização dos dados

O levantamento bibliográfico foi realizado em abril de 2023, nas bases de dados PubMed, EMBASE, *Cumulative Index to Nursing and Allied Health Literature* (CINAHL), Literatura Latino-Americana e do Caribe em Ciências da Saúde (LILACS) e PsycINFO. Visando contemplar o maior número de estudos, foram escolhidos descritores apropriados para as bases de dados, assim como foram utilizadas palavras-chave. No Quadro 1 encontram-se as estratégias de buscas, salienta-se que as mesmas foram construídas por bibliotecária com expertise na área da saúde. Para a recuperação dos estudos, foram aplicados filtros elaborados por expertises em informação da *BMJ Knowledge Center* ^(17)^ e da *InterTASC Information Specialists’ Sub-Group* (ISSG)^(18)^. Também foram seguidas as recomendações de elaboração de estratégias de busca preconizadas pela *Peer Review of Electronic Search Strategies* (PRESS)^(19)^.

**Quadro 1:** Estratégias de busca nas bases de dados, 2023

Base de dados	Estratégia de busca
PubMed	*((“analgesia”[MeSH Terms] OR “analgesia”[All Fields] OR “analgesias”[All Fields] OR “pain relief”[Text Word] OR (“pain”[MeSH Terms] OR “pain”[All Fields]) OR (“anxiety”[MeSH Terms] OR “anxiety”[All Fields] OR “anxieties”[All Fields] OR “anxiety s”[All Fields]) OR (“fear”[MeSH Terms] OR “fear”[All Fields])) AND (equipment and supplies [tw] OR “ShotBlocker”[All Fields] OR “equipment design”[Text Word] OR “device”[Title] OR “Design”[Title])) AND (“inject”[All Fields] OR “injectability”[All Fields] OR “injectant”[All Fields] OR “injectants”[All Fields] OR “injectate”[All Fields] OR “injectates”[All Fields] OR “injected”[All Fields] OR “injectible”[All Fields] OR “injectibles”[All Fields] OR “injecting”[All Fields] OR “injections”[MeSH Terms] OR “injections”[All Fields] OR “injectable”[All Fields] OR “injectables”[All Fields] OR “injection”[All Fields] OR “injects”[All Fields] OR (“needle s”[All Fields] OR “needled”[All Fields] OR “needles”[MeSH Terms] OR “needles”[All Fields] OR “needle”[All Fields] OR “needling”[All Fields] OR “needlings”[All Fields]))*
EMBASE	*(‘equipment design’/exp OR ‘equipment design’ OR ‘product design’/exp OR ‘product design’ OR ‘shotblocker’) AND (‘injection’/exp OR ‘blood vessel injection’ OR ‘gluteal injection’ OR ‘injection’ OR ‘injection solution’ OR ‘injections’ OR ‘percutaneous injection’ OR ‘needle’/exp) AND (‘fear’/exp OR ‘fear’ OR ‘anxiety’/exp OR ‘analgesia’/exp OR ‘analgesia’ OR ‘analgesia’ OR ‘pain management’ OR ‘pain relief’ OR ‘sequential analgetic analgesia’ OR ‘surgical analgesia’ OR ‘pain’/exp OR ‘acute pain’ OR ‘deep pain’ OR ‘lightning pain’ OR ‘nocturnal pain’ OR ‘pain’ OR ‘pain response’ OR ‘pain syndrome’ OR ‘treatment related pain’)*
CINAHL LILACS PSYCINFO	*(analgesia OR pain relief OR pain OR anxiety OR fear) AND (injection OR needle) AND (equipment design OR devices OR design OR equipment and supplies OR shotblocker)*

### Análise dos dados

A seleção dos estudos ocorreu em duas etapas, sendo a primeira a leitura dos títulos e resumos por dois juízes independentes, por intermédio da Ferramenta *On-line Rayyan QCRI* ^(20)^. Nos casos em que ocorreu discordância entre os juízes, foi convocado um terceiro juiz para avaliar e decidir pela inclusão ou exclusão do estudo. Na segunda etapa, um juiz separou os estudos pré-selecionados e os enviou na íntegra para os demais juízes. De forma independente e considerando o objetivo dessa revisão, cada juiz avaliou e optou pela inclusão ou exclusão do estudo, seguindo os critérios preestabelecidos. Nos casos em que não houve consenso, o terceiro juiz foi novamente consultado.

A seleção final foi composta por estudos que apontam a utilização de dispositivos de pressão local no alívio da dor associada às injeções subcutânea, intradérmica e intramuscular em pacientes independentes da faixa etária. Os estudos foram caracterizados a partir de uma análise descritiva, realizada por meio de instrumento de coleta elaborado pelos autores para extrair e delimitar as evidências. O instrumento de coleta contemplou aspectos relacionados à caracterização da publicação (autoria, ano, país), objetivo, características metodológicas (desenho do estudo, amostra, cenário), via de administração, principais resultados e nível de evidência do estudo.

Para classificar o nível de evidência dos estudos foi utilizada a categorização preconizada pela *Agency for Healthcare Research and Quality* (AHRQ)^(21)^.

Para sumarizar os resultados referente a utilização dos dispositivos de pressão local no alívio da dor associada a injeções subcutânea, intradérmica e intramuscular foi utilizado quadro, como recomenda o JBI, para facilitar a sua visualização.

## RESULTADOS

Nas bases de dados foram encontrados 1.514 estudos. Durante a primeira avaliação, 188 estudos foram excluídos por estarem duplicados, restando 1.326 para leitura de título e resumo. Nesta etapa, 1.295 estudos foram removidos por não atenderem ao propósito desta revisão. Com isso, 31 estudos foram selecionados para leitura na íntegra e análise de elegibilidade, sendo 11 estudos excluídos. A amostra foi composta por 20 artigos, sendo o processo de identificação, seleção, elegibilidade e inclusão descritos na Figura 1, conforme o fluxograma *Preferred Reporting Items for Systematic Reviews and Meta-Analyses* (PRISMA)^(22)^.

**Figura 1:** Fluxograma do processo de seleção de artigos da revisão, 2023

*Fonte: Adaptado do PRISMA^(22)^*

Dentre os 20 artigos incluídos, os mais antigos foram publicados em 2009 e o mais recente em 2023. Sendo dois (10%) publicados em 2009, um (5%) em 2015, dois (10%) em 2017, um (5%) em 2018, cinco (25%) em 2019, um (5%) em 2020, quatro (20%) em 2021, três (15%) em 2022 e um (5%) em 2023. Logo, 70% das publicações incluídas nesta revisão são dos últimos cinco anos (2019 a março de 2023).

Quanto ao país de origem dos estudos, a Turquia liderou as pesquisas acerca do alívio da dor associada à injeção com 16 (80%) estudos e os EUA foram responsáveis por dois (10%) estudos, enquanto o Reino Unido e a Índia foram responsáveis por um (5%) estudo cada. No que concerne ao delineamento dos estudos, onze (55%) são ensaios clínicos/estudos experimentais, quatro (20%) são estudos prospectivos, dois (10%) são quase experimentais, dois (10%) são revisões sistemáticas e um (5%) é uma revisão de literatura.

Quanto aos dispositivos de pressão local, 20 (100%) estudos contemplam o ShotBlocker^®^. Salienta-se que os estudos com o Pikluc^®^ são escassos e que não foram resgatados nas bases de dados por não estarem em material indexado. No entanto, esse dispositivo faz parte da realidade brasileira e vem sendo utilizado no cuidado à saúde. Referente à via de administração, 16 (80%) estudos contemplam a via intramuscular, três (15%) a via subcutânea e um (5%) aborda simultaneamente as vias intramuscular e subcutânea.

Os estudos incluídos nesta revisão foram apresentados no Quadro 2, contendo informações quanto à autoria, ano, país, objetivo, desenho do estudo, amostra, cenário, via de administração, principais resultados e nível de evidência.

**Quadro 2:** Características dos estudos incluídos na revisão de escopo, 2023

ID^*^	Autoria Ano País	Objetivo	Desenho do estudo Amostra Cenário Via de administração	Principais resultados	Nível de Evidência
E1	Cobb *et al.* ^(23)^ 2009 Estados Unidos da América	Examinar a eficácia do ShotBlocker^®^, uma intervenção física projetada para diminuir a dor da injeção das crianças.	Tipo de estudo: Ensaio clínico randomizado e controlado. Amostra: 89 crianças, entre 4 e 12 anos de idade e seus respectivos pais. Cenário: Clínica pediátrica. Via de administração: Subcutânea e intramuscular.	Não houve evidências quanto à eficácia do ShotBlocker^®^ na redução da dor associada às injeções de imunizantes comuns na infância. Assim, como não foi eficaz na redução da ansiedade infantil.	II
E2	Drago *et al.* ^(24)^ 2009 Estados Unidos da América	Determinar a eficácia do ShotBlocker^®^ na redução da dor pediátrica em injeções intramusculares.	Tipo de estudo: Estudo prospectivo randomizado e controlado. Amostra: 165 crianças, entre 2 meses e 17 anos de idade. Cenário: Ambulatório pediátrico de Hospital Universitário. Via de administração: Intramuscular.	A dor percebida pelos enfermeiros e responsáveis teve escores mais baixos nas crianças que utilizaram o ShotBlocker^®^ durante a imunização. No entanto, essa eficácia não foi tão evidente no autorrelato das crianças de 36 meses ou mais quanto a sua dor. Contudo, as crianças com 72 meses ou mais relataram alívio da dor ao utilizar o ShotBlocker^®^.	II
E3	Çelik *et al.* ^(25)^ 2015 Turquia	Avaliar a efetividade do ShotBlocker^®^ na redução da dor e ansiedade associada à injeção intramuscular em adultos.	Tipo de estudo: Ensaio clínico randomizado, controlado por placebo. Amostra: 180 adultos, entre 18 e 80 anos de idade. Cenário: Ambulatório de Hospital Público. Via de administração: Intramuscular.	Os indivíduos que utilizaram o ShotBlocker^®^ durante a injeção intramuscular de diclofenaco sódico (75 mg/3 mL) obtiveram escores de dor significativamente menor quando comparado aos indivíduos dos grupos controle e placebo. Logo, o ShotBlocker^®^ é recomendado para aliviar a dor associada a injeção intramuscular em adultos. Contudo, não reduziu a ansiedade. Além disso pode causar ansiedade adicional, sendo essencial a apresentação do dispositivo aos indivíduos antes de seu uso.	II
E4	Caglar *et al.* ^(26)^ 2017 Turquia	Examinar a eficácia do ShotBlocker^®^ para controlar a dor da injeção associada à primeira vacina intramuscular contra Hepatite B administrada em recém-nascidos saudáveis a termo.	Tipo de estudo: Estudo prospectivo randomizado controlado. Amostra: 100 recém-nascidos saudáveis a termo. Cenário: Enfermaria de Hospital Universitário privado. Via de administração: Intramuscular.	Os escores de dor a partir da *Neonatal Infant Pain Scale* (NIPS) no momento e pós-injeção foram significativamente menores nos neonatos que utilizaram o ShotBlocker^®^, quando comparados ao grupo controle. A frequência cardíaca foi menor no grupo intervenção três minutos após a injeção, mas não houve diferença significativa na frequência respiratória. O ShotBlocker^®^ é recomendado para aliviar a dor associada a injeção intramuscular em recém-nascidos saudáveis.	II
E5	Emel *et al.* ^(27)^ 2017 Turquia	Examinar os efeitos do ShotBlocker^®^ no alívio da dor devido à vacinação contra Hepatite B no músculo deltoide em adultos.	Tipo de estudo: Estudo randomizado, controlado, simples-cego. Amostra: 242 indivíduos, entre 18 e 31 anos de idade. Cenário: Departamentos de Enfermagem e Obstetrícia de uma Escola de Saúde (Universidade). Via de administração: Intramuscular.	Não houve diferença significativa entre os grupos experimental e controle quanto a dor associada a vacina contra Hepatite B administrada no músculo deltoide. Logo, o ShotBlocker^®^ não foi eficaz na redução da dor. Contudo, observou-se que o índice de massa corporal (IMC) interfere na percepção de dor, sendo que à medida que o IMC aumenta, a dor diminui.	II
E6	Canbulat Sahiner *et al.* ^(28)^ 2018 Turquia	Comparar o efeito do ShotBlocker^®^ e da combinação de vibração e aplicação de frio (Buzzy^®^) na redução da dor durante a administração de insulina em crianças.	Tipo de estudo: Estudo experimental randomizado controlado. Amostra: 60 crianças, entre 6 e 12 anos de idade. Cenário: Departamento de Endocrinologia Infantil de uma Faculdade de Medicina (Universidade). Via de administração: Subcutânea.	Na dor percebida pelos responsáveis e pesquisador/observador, assim como no autorrelato das crianças observou-se que as crianças do grupo controle apresentaram escores de dor mais altos quando comparados aos escores dos grupos Buzzy^®^ e ShotBlocker^®^. Os escores de ansiedade das crianças dos grupos Buzzy^®^ e ShotBlocker^®^ foram menores quando comparados ao grupo controle. As crianças que utilizaram o ShotBlocker^®^ tiveram as pontuações mais baixas tanto no quesito da dor quanto da ansiedade.	II
E7	Aydin *et al.* ^(8)^ 2019 Turquia	Examinar o efeito do ShotBlocker^®^ no alívio da dor associada à injeção intramuscular.	Tipo de estudo: Estudo controlado e quase experimental. Amostra: 50 mulheres, entre 18 e 45 anos de idade. Cenário: Unidade de internação hospitalar. Via de administração: Intramuscular.	O grupo experimental apresentou escores de dor estatisticamente significante mais baixos quando comparados ao grupo controle. Recomenda-se o uso do ShotBlocker^®^ nos serviços e protocolos de injeção intramuscular e divulgação de evidências científicas do mesmo como método não farmacológico a fim de promover o seu uso na prática clínica da Enfermagem.	III
E8	Bilge *et al.* ^(29)^ 2019 Turquia	Comparar a eficácia do ShotBlocker^®^ e do spray gelado na redução da dor relacionada à injeção intramuscular em adultos.	Tipo de estudo: Estudo prospectivo, randomizado e controlado. Amostra: 120 adultos, com idade igual ou maior que 18 anos. Cenário: Departamento de Medicina de Emergência de uma Escola de Medicina (Universidade). Via de administração: Intramuscular.	O ShotBlocker^®^ foi considerado um método não farmacológico eficaz na redução da dor relacionada à injeção intramuscular, não havendo diferença entre a sua eficácia e a do spray gelado. Contudo, administrar a injeção com o ShotBlocker^®^ foi mais difícil quando comparado ao spray gelado, segundo os participantes. Recomenda-se novos estudos para facilitar o uso do ShotBlocker^®^ na prática clínica.	II
E9	Sivri Bilgen *et al.* ^(30)^ 2019 Turquía	Investigar o efeito do Buzzy^®^ e ShotBlocker^®^ na redução da dor induzida por injeções intramusculares de penicilina em crianças.	Tipo de estudo: Estudo controlado randomizado. Amostra: 150 crianças, entre 7 e 12 anos de idade. Cenário: Clínica de emergência pediátrica. Via de administração: Intramuscular.	As crianças do grupo controle apresentaram escores de dor significativamente maiores durante a injeção de penicilina do que as crianças dos grupos ShotBlocker^®^ e Buzzy^®^. O grupo Buzzy^®^ apresentou os menores escores de dor quando comparados aos grupos ShotBlocker^®^ e controle. Contudo, o ShotBlocker^®^ apresentou escores mais baixos quando comparado ao grupo controle, sendo uma opção de método não farmacológico para alívio da dor associada a injeção intramuscular. A ansiedade avaliada antes do procedimento não teve diferença estatisticamente significante entre os grupos.	II
E10	Yilmaz *et al.* ^(31)^ 2019 Turquia	Comparar a efetividade do Buzzy^®^, ShotBlocker^®^ e bolha de sopro na redução da dor e medo associados a injeção intramuscular em crianças.	Tipo de estudo: Ensaio clínico prospectivo e randomizado. Amostra: 160 crianças, entre 5 e 10 anos de idade. Cenário: Pronto-socorro pediátrico hospitalar. Via de administração: Intramuscular.	A dor e o medo foram significativamente menores nas crianças que utilizaram o Buzzy^®^, quando comparados aos grupos controle, ShotBlocker^®^ e soprar bolhas. Contudo, as crianças do grupo ShotBlocker^®^ também obtiveram escores de dor e medo menores quando comparados aos grupos controle e soprar bolhas. Deste modo, os dispositivos ShotBlocker^®^ e Buzzy^®^ são recomendados durante a aplicação de injeção intramuscular para redução da dor e medo infantis associadas ao procedimento.	II
E11	Şanlialp Zeyrek *et al.* ^(32)^ 2019 Turquia	Avaliar a eficácia das intervenções físico-processuais na redução da dor durante injeções intramusculares.	Tipo de estudo: Revisão sistemática e meta-análise. Amostra: 15 estudos. Cenário: Não se aplica. Via de administração: Intramuscular.	O ShotBlocker^®^ é uma ferramenta a ser considerada para aliviar a dor associada a injeções intramusculares.	I
E12	Inangil *et al.* ^(33)^ 2020 Turquia	Investigar o efeito da mecanoanalgesia e da aplicação de frio na equimose, dor e satisfação do paciente por injeção subcutânea de heparina.	Tipo de estudo: Pesquisa prospectiva, controlada, quase-experimental e clínica. Amostra: 55 adultos, com idade igual ou maior que 18 anos. Cenário: Enfermarias de Ortopedia e Traumatologia de Hospital Universitário. Via de administração: Subcutânea.	Tanto o uso da analgesia mecânica por meio do ShotBlocker^®^ quanto a aplicação de frio reduziram a dor associada a injeção subcutânea de heparina. O ShotBlocker^®^ foi ineficaz na equimose, mas obteve maior nível de satisfação, com significância estatística.	III
E13	Ayinde *et al.* ^(34)^ 2021 Reino Unido	Revisar o efeito de diferentes técnicas de injeção intramuscular na dor associada à injeção, em adultos.	Tipo de estudo: Revisão sistemática e meta-análise. Amostra: 29 estudos. Cenário: Não se aplica. Via de administração: Intramuscular.	Observou-se evidências contraditórias quanto a eficácia do ShotBlocker^®^, no qual um estudo evidencia que houve redução da dor associada a injeção intramuscular, enquanto outro estudo atesta que a dor foi semelhante no grupo controle e intervenção. Ressalta-se que a diferença entre os resultados pode estar relacionada à maior população de estudo, menor volume de injeção e grupo de pacientes mais saudáveis.	I
E14	Kolcu *et al.* ^(35)^ 2021 Turquia	Investigar o efeito do ShotBlocker^®^ nos níveis de dor, ansiedade e satisfação na injeção subcutânea de pacientes com urticária crônica espontânea.	Tipo de estudo: Ensaio clínico randomizado e controlado. Amostra: 90 indivíduos, com idade igual ou maior que 18 anos. Cenário: Clínica de dermatologia de Hospital Universitário. Via de administração: Subcutânea.	Quanto a dor e ao estado de ansiedade, não houve diferença estatisticamente importante entre os grupos ShotBlocker^®^, placebo e controle na avaliação após a injeção. Quanto ao nível de satisfação após a injeção, o grupo ShotBlocker^®^ teve um resultado estatisticamente alto quando comparado aos grupos placebo e controle. Contudo, acredita-se que o ShotBlocker^®^ possa ser apresentado aos pacientes como uma opção por ser de fácil e econômico uso.	II
E15	Şahan *et al.* ^(36)^ 2021 Turquia	Determinar os efeitos da aplicação do ShotBlocker^®^ durante a administração de injeções intramusculares em pacientes adultos para fornecer uma prática baseada em evidências.	Tipo de estudo: Revisão da literatura com meta-análise. Amostra: 5 estudos. Cenário: Não se aplica. Via de administração: Intramuscular.	Os níveis de dor no grupo experimental em que ShotBlocker^®^ foi usado durante a injeção intramuscular em pacientes adultos foram significativamente diferentes em comparação com o grupo controle. Como resultado da meta-análise, descobriu-se que a aplicação do ShotBlocker^®^ na injeção intramuscular em pacientes adultos reduziu a intensidade da dor dos pacientes.	I
E16	Yildirim *et al.* ^(37)^ 2021 Turquia	Avaliar o efeito do ShotBlocker^®^ na dor e na satisfação da injeção intramuscular em pacientes adultos de emergência.	Tipo de estudo: Ensaio clínico randomizado. Amostra: 74 indivíduos, com idade igual ou maior que 18 anos. Cenário: Pronto-socorro adulto hospitalar. Via de administração: Intramuscular.	O nível de dor no grupo ShotBlocker^®^ foi menor, quando comparado ao grupo controle. Enquanto o nível de satisfação do grupo ShotBlocker^®^ foi maior quando comparado ao grupo controle. Em ambos os casos, a diferença foi estatisticamente significante.	II
E17	Gürdap *et al.* ^(38)^ 2022 Turquia	Comparar os efeitos do spray frio e ShotBlocker^®^ na redução da dor em adultos causada por injeção intramuscular no pronto-socorro adulto.	Tipo de estudo: Ensaio clínico randomizado e controlado. Amostra: 195 indivíduos, com idade igual ou maior que 18 anos. Cenário: Pronto-socorro adulto de Hospital Universitário. Via de administração: Intramuscular.	O spray frio mostrou-se como o melhor recurso para manejar a dor associada a injeção intramuscular. No entanto, o ShotBlocker^®^ também é uma opção para redução da dor, uma vez que, mostrou-se eficaz quando comparado com o grupo controle e aos grupos placebos.	II
E18	Savcı *et al.* ^(39)^ 2022 Turquia	Examinar a eficácia do ShotBlocker^®^ e vibração local na percepção de dor e satisfação durante a injeção intramuscular de antibiótico.	Tipo de estudo: Estudo experimental randomizado controlado. Amostra: 100 indivíduos, com idade igual ou maior que 18 anos. Cenário: Pronto-socorro adulto hospitalar. Via de administração: Intramuscular.	A aplicação de vibração local foi mais eficaz na redução da dor e no aumento da satisfação durante a injeção intramuscular de antibióticos quando comparado aos grupos ShotBlocker^®^ e controle. Nota-se que os escores de dor foram menores e o de satisfação maiores no grupo ShotBlocker^®^ quando comparado ao controle.	II
E19	Zengin *et al.* ^(40)^ 2022 Turquia	Testar uma nova intervenção não farmacológica para reduzir a dor relacionada à agulha no departamento de emergência pediátrica.	Tipo de estudo: Ensaio clínico randomizado e controlado. Amostra: 159 crianças, entre 7 e 10 anos de idade. Cenário: Emergência pediátrica. Via de administração: Intramuscular.	Na dor percebida pelos responsáveis e pesquisador/observador, assim como no autorrelato das crianças observou-se que os escores de dor do grupo *Palm Stimulator* foram estatisticamente e significativamente menores quando comparados aos grupos ShotBlocker^®^ e controle. Embora a percepção dos níveis de dor das crianças no grupo ShotBlocker^®^ sejam menores que as do grupo controle, esta diferença não foi estatisticamente significante. Quanto ao medo pré-procedimento, não houve diferença entre os grupos.	II
E20	Cmc *et al.* ^(41)^ 2023 Índia	Sintetizar as melhores evidências de pesquisa disponíveis sobre a eficácia da estimulação física para reduzir a dor em adultos recebendo injeções intramusculares.	Tipo de estudo: Revisão sistemática e meta-análise. Amostra: 25 estudos. Cenário: Não se aplica. Via de administração: Intramuscular.	O ShotBlocker^®^ é eficaz na redução da dor associada a injeção intramuscular. Contudo, observa-se que o dispositivo pode aumentar o nível de ansiedade dos pacientes. Reforça-se que é uma técnica promissora no manejo da dor associada a injeção intramuscular, com baixo custo, além de poder ser facilmente ensinada/aprendida.	I

* *ID = Identificação do estudo.*

## DISCUSSÃO

Este trabalho corrobora para o avanço da investigação acerca da utilização dos dispositivos de pressão local ShotBlocker^®^ e Pikluc^®^ durante a aplicação de injeções, visando diminuir a dor, a ansiedade e o medo associados a estes procedimentos. Contudo, nota-se que ao realizar a busca nas bases de dados, os resultados encontrados tanto em nível nacional quanto internacional, contemplam apenas o dispositivo ShotBlocker^®^ e que o único estudo realizado com Pikluc^®^ até então, não foi resgatado nas bases de dados. Todavia, os resultados evidenciados nesta revisão indicam pontos convergentes e divergentes quanto aos benefícios do uso do dispositivo ShotBlocker^®^ durante a aplicação de injeções intramuscular e subcutânea em pacientes no contexto da assistência à saúde.

Durante o período neonatal, o recém-nascido (RN) é submetido a diferentes procedimentos dolorosos, incluindo a vacina contra Hepatite B por via intramuscular (E4)^(26)^. O fenômeno da dor na Neonatologia é subjetivo e complexo, além de ser impossível o relato verbal por parte do RN, sendo necessário considerar parâmetros comportamentais e fisiológicos para se obter uma avaliação multidimensional da dor^(42)^. Ao utilizar o dispositivo ShotBlocker^®^ em RN a termo e saudável, observa-se um efeito positivo na redução da dor associada à vacinação, atestada pela *Neonatal Infant Pain Scale* (NIPS), assim como um efeito sistêmico ao reduzir a frequência cardíaca do neonato. Contudo, o ShotBlocker^®^ não interferiu na frequência respiratória dos RNs^(26)^.

O ShotBlocker^®^ é um dispositivo à disposição da Enfermagem, de fácil acesso e com custo acessível que se mostrou eficaz na redução da dor em neonatos. Todavia, reconhece-se que a frequência cardíaca representa uma resposta fisiológica, enquanto a escala NIPS fornece uma resposta comportamental à dor e não a intensidade da mesma. Além disso, esse foi o primeiro procedimento com agulha experienciado pelos RNs participantes do estudo e sabe-se que a resposta à dor também está relacionada a experiências pregressas (E4)^(26)^.

Na Pediatria, o ShotBlocker^®^ foi utilizado em diferentes faixas etárias, compreendendo dos dois meses aos 17 anos de idade e, vias de administração, intramuscular e/ou subcutânea (E1, E2, E6, E9, E10, E19)^(23-24,28,30-31,40)^. Ao se avaliar a dor de crianças durante a imunização de rotina por via intramuscular, por meio da Escala Baker-Wong FACES, constatou-se que o autorrelato das crianças entre 36 e 71 meses de idade não evidenciou um efeito positivo do dispositivo ShotBlocker^®^, porém quando avaliado o autorrelato de crianças a partir dos 72 meses ou mais, nota-se que o dispositivo foi útil na redução da dor associada à injeção intramuscular, corroborando com a avaliação dos responsáveis e dos enfermeiros que acompanharam o procedimento (E2)^(24)^. Apesar de não terem sido considerados e avaliados outros parâmetros que permeiam a dor.

Durante a administração de penicilina por via intramuscular em crianças entre sete e 12 anos de idade, buscou-se comparar os dispositivos ShotBlocker^®^ e Buzzy^®^ (E9)^(30)^. O Buzzy^®^ é um dispositivo que associa a vibração com a aplicação do gelo, sendo recomendado durante a realização de procedimentos com agulhas^(43)^. Para mensuração da dor, foram utilizadas as Escalas *Visual Analog Scale* (VAS) e *Faces Pain Scale-Revised* (FPS-R), além de ser aplicada antes do procedimento para mensurar a ansiedade das crianças a Escala *State-Trait Anxiety Inventory for Children* (STAIC)^(30)^. Quanto à ansiedade, não houve diferença entre os grupos, mas quanto à dor observou-se que o Buzzy^®^ apresentou os melhores resultados, apesar do ShotBlocker^®^ também se mostrar eficaz quando comparado ao grupo controle (E9)^(30)^.

Ao se comparar o ShotBlocker^®^, o Buzzy^®^ e a bolha de sopro no alívio da dor associada à injeção intramuscular em crianças entre cinco e dez anos de idade, por meio dos escores de dor de Oucher^(31)^, observou-se que o Buzzy^®^ apresenta os melhores resultados como evidenciado no estudo anterior. No entanto, o ShotBlocker^®^ apresenta escores de dor menores quando comparado com a bolha de sopro e o grupo controle. Esse resultado foi constatado também na avaliação do medo das crianças por meio da Escala *Children’s Fear Scale* (E10)^(31)^. Em suma, apesar do Buzzy^®^ ser mais eficaz na redução da dor e do medo, o ShotBlocker^®^ ainda é uma opção de dispositivo para diminuir a dor e o medo associados a injeções intramusculares. Reforça-se que os mecanismos de ação dos dispositivos também são diferentes.

Outro dispositivo que foi comparado ao ShotBlocker^®^ na Pediatria foi o *Palm Stimulator* ^(40)^. Neste estudo, crianças entre sete e dez anos de idade submetidas a injeção intramuscular foram avaliadas quanto à dor pelas Escalas VAS e FPS-R e para o medo utilizou-se a Escala *Children’s Fear Scale*. A dor avaliada pela criança, responsável e pesquisador obteve escores menores no grupo *Palm Stimulator* ^(40)^. Porém, diferentemente dos demais estudos, o ShotBlocker^®^ não obteve diferença estatisticamente significante quando comparado ao grupo controle, apesar de ter escore de dor menores. Em relação ao medo, avaliado antes do procedimento, não houve diferença entre os grupos. Salienta-se que tal dado é relevante para a avaliação da dor, uma vez que, o medo pode interferir na resposta da criança (E19)^(40)^.

Quanto à via subcutânea em crianças, o ShotBlocker^®^ foi testado em dois estudos (E1, E6)^(23,28)^. No estudo desenvolvido durante a aplicação de imunizantes por via subcutânea e intramuscular em crianças entre quatro e 12 anos de idade, observou-se que o ShotBlocker^®^ não foi eficaz na redução da dor e da ansiedade infantil^(23)^. Para a avaliação da dor pela criança foi utilizado o autorrelato por meio da Escala FPS-R, enquanto os responsáveis e pesquisador utilizaram a Escala VAS. Coube ainda aos responsáveis e pesquisador observarem o comportamento/angústia apresentado pelas crianças antes, durante e após o procedimento, além de avaliar a ansiedade por meio da Escala VAS (E1)^(23)^. Reforça-se que esta escala não foi desenvolvida com o propósito de avaliar a ansiedade, mas a dor.

Em contraste com estudos anteriores, ao comparar o ShotBlocker^®^ e o Buzzy^®^ durante a aplicação de insulina em crianças entre seis e 12 anos de idade diagnosticadas com Diabetes Mellitus Tipo 1, o ShotBlocker^®^ obteve os menores escores tanto na avaliação da dor quanto da ansiedade, a partir do autorrelato, da avaliação dos responsáveis e do pesquisador^(28)^. Contudo, o Buzzy^®^ também foi efetivo, quando comparado ao grupo controle. Neste estudo, foi utilizado um formulário de entrevista e observação, além das Escalas *Children’s Anxiety and Pain Scale* (CAPS) e FPS-R (E6)^(28)^. Frisa-se que este resultado pode estar relacionado a via de administração, pois a via subcutânea requer técnica mais simples e menos dolorosa quando comparada à injeção por via intramuscular^(44)^.

No contexto da assistência ao adulto, o ShotBlocker^®^ também foi avaliado durante a administração de medicamento pela via subcutânea, sendo as medicações heparina (E12)^(33)^ e omalizumabe (E14)^(35)^. Estudo buscou comparar o efeito do ShotBlocker^®^ e da aplicação de frio durante a administração de heparina na região abdominal em pacientes a partir dos 18 anos de idade^(33)^. Para mensurar a dor e a satisfação do paciente foi utilizada a Escala VAS, além de uma Ferramenta para avaliar a equimose. Observou-se que o ShotBlocker^®^ obteve os melhores resultados no quesito dor e satisfação, no entanto, não teve efeito sobre a equimose. Ressalta-se que quanto menor a dor sentida pelo paciente, maior será a sua satisfação (E12)^(33)^.

O ShotBlocker^®^ foi efetivo durante a administração de injeção por via subcutânea, tanto no público pediátrico quanto adulto. Contudo, apesar dos resultados semelhantes, destaca-se que no estudo conduzido com as crianças foi utilizado o dispositivo Buzzy^®^ que associa o frio com a vibração (E6)^(28)^, enquanto com os adultos foi utilizada apenas a aplicação do frio (E12)^(33)^.

Durante a aplicação de omalizumabe por via subcutânea em indivíduos com idade igual ou superior a 18 anos, notou-se que o ShotBlocker^®^ não apresentou diferença estatística significante na redução da dor e da ansiedade, quando comparado aos grupos controle e placebo, sendo que este utilizou a superfície lisa do dispositivo^(35)^. Contudo, os participantes ficaram mais satisfeitos com o uso do dispositivo. Assim como no estudo anterior, foi utilizada a Escala VAS para avaliação da dor e da satisfação, além do *State-Trait Anxiety Inventory Form* para mensuração da ansiedade (E14)^(35)^. Constata-se uma variação quanto ao efeito do ShotBlocker^®^ na redução da dor durante a injeção subcutânea, podendo esse resultado estar relacionado aos diferentes locais de aplicação, bem como à substância administrada.

Quanto ao uso do ShotBlocker^®^ em adultos durante a aplicação de injeção intramuscular, observa-se um maior número de estudos (E3, E5, E7, E8, E16, E17, E18)^(8,25,27,29,37-39)^. O ShotBlocker^®^ foi eficaz na redução da dor durante a aplicação de diclomec 12 horas pós-cirúrgico (E7)^(8)^ e diclofenaco sódico (E3, E16)^(25,37)^. Porém, em estudo semelhante, observou-se que o spray frio foi mais eficaz na redução da dor durante a aplicação de diclofenaco sódico do que o ShotBlocker^®^ (E17)^(38)^. Assim como, a vibração local foi mais eficaz durante a administração de antibióticos (E18)^(39)^. Contudo, o ShotBlocker^®^ continua sendo uma opção viável para o manejo da dor segundo estes mesmos estudos (E17, E18)^(38-39)^.

Em contraste ao encontrado em estudo anterior, durante a administração de diclofenaco sódico não houve diferença estatisticamente significativa quando utilizado o ShotBlocker^®^ ou o spray frio^(29)^. Inclusive, neste estudo constatou-se que os profissionais sentiram dificuldade em aplicar a injeção intramuscular utilizando o ShotBlocker^®^ (E8)^(29)^. Apenas um estudo na população adulta afirmou que o ShotBlocker^®^ não é eficaz na redução da dor, tendo como cenário a administração da vacina contra Hepatite B em indivíduos com idade entre 18 e 31 anos (E5)^(27)^, indo de encontro aos resultados constatados na Neonatologia (E4)^(26)^.

A Escala VAS foi utilizada para mensurar a dor (E3, E5, E7, E8, E16, E17, E18)^(8,25,27,29,37-39)^ e a satisfação dos pacientes (E16, E18)^(37,39)^, enquanto o *State-Trait Anxiety Inventory Form* visou avaliar a ansiedade (E3)^(25)^. Quanto à satisfação dos participantes acerca do uso do ShotBlocker^®^ durante a injeção intramuscular, observa-se uma resposta positiva (E16, E18)^(37,39)^. Contudo, em um estudo verificou-se que o dispositivo pode aumentar o nível de ansiedade do paciente, porém ainda é indicado como um recurso a ser implementado na prática clínica (E3)^(25)^.

### Limitações do estudo

Essa pesquisa possui importantes limitações, são elas: a carência de estudos com o dispositivo brasileiro Pikluc^®^; a inexistência de estudos com a utilização dos dispositivos em injeção intradérmica; a ausência de referências cruzadas que pudessem ser incluídas na amostra; e a possível perda de estudos devido à não utilização de descritores controlados por algumas bases de dados.

### Contribuições para a área da Enfermagem

Os achados desta revisão trazem importantes contribuições, pois possibilitam discussões sobre o manejo da dor, da ansiedade e do medo durante a aplicação de injeções subcutânea e intramuscular no público pediátrico e adultos. Além disso, contribuem para a disseminação do conhecimento acerca dos dispositivos de pressão local, bem como seus benefícios e suas limitações, e corroboram para o aprimoramento dos cuidados de Enfermagem na prática clínica durante a realização de procedimentos dolorosos e pontuais.

## CONSIDERAÇÕES FINAIS

O dispositivo de pressão local ShotBlocker^®^ é uma tecnologia disponível para a Enfermagem durante a realização de procedimentos dolorosos, como as injeções subcutânea e intramuscular. Observa-se que esse recurso, de baixo custo e fácil manuseio, pode ser útil no manejo da dor, da ansiedade e do medo tanto em pacientes pediátricos quanto adultos. É notável o número de investigações que demonstram a utilização do ShotBlocker^®^ na prática clínica, contudo, ainda há divergências na literatura quanto à sua efetividade. Deste modo, é essencial a realização de estudos clínicos controlados que considerem variáveis como idade, índice de massa corporal, medicação, calibre da agulha, dentre outras, e que a comparação ocorra com outros dispositivos que utilizem o mesmo mecanismo de ação. Recomenda-se ainda a realização de estudos com o dispositivo Pikluc^®^ no âmbito nacional e internacional, bem como estudos que investiguem o uso de ambos os dispositivos na via intradérmica.
